# The timing of administering aspirin and nitroglycerin in patients with STEMI ECG changes alter patient outcome

**DOI:** 10.1186/s12873-021-00523-2

**Published:** 2021-11-17

**Authors:** Kristijan B. Todoroski

**Affiliations:** Family medicine department at univ. St. Kiril and Methodius in Skopje, Republic of North Macedonia, employed at Private family medicine clinic “Azura”, Skopje, North Macedonia

## Abstract

**Background:**

Both chewed aspirin and sublingual nitroglycerin are fast acting medications and reach therapeutic levels within a few minutes. Current guidelines for managing acute coronary syndrome (ACS) do not recognize the importance of the order or timing of administering aspirin and nitroglycerin. This retrospective study aimed to examine if there was any benefit to the timing of giving aspirin before or after nitroglycerin in cases of ACS.

**Methods:**

From the large National Emergency Medical Services Information System (NEMSIS) 2017 Version database, 2594 patients with acute coronary syndrome were identified (based on chest pain and their ECG finding) that received aspirin plus nitroglycerin in prehospital settings. Based on which medication was given first, the patients were separated in 2 groups: an aspirin-first and a nitroglycerin-first group. The 2246 patients who received aspirin first were further stratified based on the time between administration of aspirin and the first dose of nitroglycerin. The other 348 patients who received nitroglycerin first were similarly stratified.

**Results:**

In patients with STEMI ischemia, giving nitroglycerin 10 min after aspirin dosing (compared to giving them simultaneously) leads to a greater than 20% reduction in need for additional nitroglycerin, a greater than 7% decrease in subjective pain experienced by the patient and reduced need for additional opioids. The aspirin-first group in total, had a 39.6% decrease in subjective pain experience after giving additional nitroglycerin compared to nitroglycerin-first group.

**Conclusion:**

In patients with ACS, this study found that giving nitroglycerin 10 min after aspirin was associated with a reduction in subjective pain scores, as well as a reduced need for additional nitroglycerin or opioids. Future prospective trials examining the timing of aspirin vs. nitroglycerin are needed to confirm these findings.

**Supplementary Information:**

The online version contains supplementary material available at 10.1186/s12873-021-00523-2.

## Introduction

Acute coronary syndrome (ACS) is a medical emergency that includes ST-elevation myocardial infarction (STEMI), non-ST elevation myocardial infarction (NSTEMI), and unstable angina. ACS is a cause of significant morbidity and mortality in the world, and is responsible for one-third of the total deaths in adults over 35 years of age worldwide. The first minutes and hours of treating ACS are of the highest importance because the life of the patient and his well-being depend of the actions that are taken initially [1–3].

In the guidelines from American College of Cardiology (ACC) and the American Heart Association (AHA) it is stated that it is wise to give aspirin and nitroglycerin in patients with acute coronary syndrome. Even though the validity of the classical MONA (morphine, oxygen, nitroglycerine and aspirin) therapy in the emergency department (ED) treatment of patients with ACS has begun to be questioned [4], aspirin plus nitroglycerin is still given in pre-hospital settings around the world. The guideline recommends that the patient with acute coronary syndrome should receive aspirin and a maximum of three doses of nitroglycerin, five minutes apart. However, the current guidelines do not recognize the importance of the order in which these medications should be given [4, 5].

This study hypothesizes that the order of administering these drugs is important, and giving aspirin several minutes before nitroglycerin can lead to a better patient outcome and theoretically can help in re-vascularization.

In support of this Gawaz [6] explains that during acute coronary syndrome the atherosclerotic plaque ruptures and thrombus is formed. This thrombus does not always lead to complete occlusion of the blood vessel and in some cases the blood circulation breaks the thrombus and leads to micro embolization of more distal arteries. The new techniques of visualization show that these events are relatively common.

Nitroglycerin causes vasodilation of the blood vessels including the artery around the thrombus, and in theory may potentiate the findings described by Gawaz [6]. However, the effect of nitroglycerin is temporary and this blood flow may re-occlude with the propagation of the thrombus. Hypothetically if aspirin is chewed and present in the circulation in a short period of time before nitroglycerin (5 to 10 min), it will manifest its anti-aggregatory effect on the thrombocytes and this may prevent the propagation of the thrombus and re-occlusion of the blood vessel. This may theoretically lead to revascularization as explained by Gawaz [6].

The hypothesis is additionally supported by the qualities of both aspirin and sublingual nitroglycerin. This will be additionally elaborated in the discussion section.

## Materials and methods

This is a retrospective cohort study on archived clinical data. The data were obtained from the NEMSIS dataset (National Emergency Medical Services Information System) 2017 Version 2018.10032, created on December 14, 2018. NEMSIS is a project designed and funded by the US government in order to standardize the reports from events in Emergency Medical Services (EMS) in the USA. The records are collected in real time by the medical staff on site, during the medical emergency [7, 8]. All data in this database is checked for double entries, completeness, logical consistency and formatting. Any data that does not pass this process are rejected and not included in the database [9].

Inclusion criteria for the patients were:

- patients that received aspirin plus nitroglycerin.

- patients with ECG findings for anterior, inferior, lateral and posterior STEMI and NON-STEMI ischemia (not all NON-STEMI patients are confirmed by troponin levels, but with ECG changes, chest pain and clinical assessment of the emergency personnel).

- patients with chest pain.

From the large NEMSIS database 9,222,789 unique emergency medical entries were identified. From these entries, specially written software in Python extracted 347,992 unique patients who received aspirin plus nitroglycerin regardless of the initial diagnosis. With additional filtering for ECG codes and presence of chest pain, we obtained 2594 final cases that had all inclusion criteria (See Supplementary 1, 9). Therefore, the final data was a group of patients suggestive for ACS confirmed by ECG findings, chest pain, medications they received and clinical assessment of the emergency personnel.

All data were divided into two main groups. Patients that received aspirin first and then nitroglycerin were the aspirin-first group (A-group) and patients that received nitroglycerin first and aspirin second were the nitroglycerin group (N-group). A system of codes was assigned for every event within the groups. A-group with 2246 patients was further stratified in 9 groups (A1 to A9) based on the time between administration of aspirin and the first dose of nitroglycerin (NTG).

The groups created are shown in Table 1.

**Table 1:** All groups created in the study for stratification and analysis

Group	Explanation
A1	NTG given 1st minute after aspirin
A2	NTG given between 1st and 2nd minute after aspirin
A3	NTG given between 2st and 3rd minute after aspirin
A4	NTG given between 3rd and 4th minute after aspirin
A5	NTG given between 4th and 5th minute after aspirin
A6	NTG given between 5th and 7th minute after aspirin
A7	NTG given between 7th and 9th minute after aspirin
A8	NTG given between 9th and 12th minute after aspirin
A9	All NTG given 12th minute after aspirin
N1	2nd NTG given within 2^t^ minutes after the first (or aspirin was given)
N2	2nd NTG (or aspirin) given between 2 and 4 min after the first NTG
N3	2nd NTG (or aspirin) given between 4 and 6 min after the first NTG
N4	2nd NTG (or aspirin) given between 6 and 8 min after the first NTG
N5	All cases where 2nd NTG (or aspirin) was given after 8 min after the first NTG

N-group with 348 patients, is different in the way that nitroglycerin is given first and aspirin second. However, in 36 cases it was found that some patients received more than 1 nitroglycerin before aspirin. These additional nitroglycerin doses were manually added as additionally administered nitroglycerin in the N-first group, because the Python software was excluding them. (See Supplementary 6).

This code system also included the data for the subjective feelings of the patients after receiving each medication, with values of: feeling better (+), feeling same (0) and feeling worse (−). This data of the subjective feeling of the patient was recorded in real time by medical professionals on site.

All of the above groups were further stratified based on how much additional nitroglycerin the patients received.

In all groups, if the patient was feeling better after administration of any medication, the software added the better (+) sign. For example, A1+ means that the patient received aspirin and within 1 min he received nitroglycerin and was feeling better. If the patient received a second dose of nitroglycerin and the patient was feeling the same the code was A1 + 20. For a third dose of nitroglycerin if the patient was feeling better: A1 + 203+ etc. This way all groups were additionally stratified in additional 5 groups based on how many additional nitroglycerin dosages were given after the first one (1 Nitro, 2 Nitro, 3 Nitro, 4 nitro, All rest).

A-group and N-group were analyzed and compared between each other. Two sets of analysis were used.

The first set analyzes the administration of additional doses of nitroglycerin after the first dose. The rationale was that if the patient is objectively feeling better the emergency service (EMS) personnel will not give any additional doses of nitroglycerin, therefore reducing the risk of unnecessary hypotension. The second analysis was on the subjective feelings of the patients. The question was: are patients feeling “better” (+) or the “same” (0) after receiving the medication. Feeling worse (−) was excluded because of insufficient data. All of the data was analyzed statistically and validated using Chi squared, paired t-test.

### Additional testing was done on the vital signs of the patients to confirm the validity of the first 2 sets of analysis. Also, additionally we did the same 2 set of tests on the exclusive STEMI group

These 2 sets of analysis were done using Microsoft Excel 16 ver. 16.0.4393.1000 with NumXL add-on ver. 1.63.42402.1 and LibreOffice 4.1.6 (See Supplementary 2, 3, 7, 8).

## Results

We analyzed a total of 2594 patients, divided in 2 groups. A-first group contained 2246 patients and N-first group 348 patients, forming a time series of data. Patients were predominantly male 69.12%; vs 30.87% female. Average age was 63.72 years. Descriptive data are presented in Table 2.

**Table 2:** Descriptive data

	Number of patients	Percentage
**All patients**	2594.00	100.00%
**Aspirin first group**	2246.00	86.58%
**NTG first group**	348.00	13.42%
**Males**	1789.00	69.12%
**Females**	799.00	30.87%
**Average age**	63.72	
**STEMI patients**	2404.00	92.71%
**NON-STEMI patients**	189.00	7.28%

Descriptive data on how different groups were formed are presented in Table 3. All figures presented were tested using Chi square of independence and Paired t test.

**Table 3:** Descriptive data for both A-First and N-First groups

	1st Nitro			2nd Nitro			3rd Nitro			All additional			SUM		
	(+)	(o)	(−)	(+)	(o)	(−)	(+)	(o)	(−)	(+)	(o)	(−)	(+)	(o)	(−)
**A1 SUM**	222 – (47.7%)			123 – (26.4%)			104 – (22.3%)			16 – (3.4%)			465 – (100%)		
**Feeling**	127	91	4	150	93	3	186	126	0	47	20	1	510	330	7
**A2 SUM**	146 – (47.4%)			94 – (30.5%)			56 – (18,1%)			12 – (3.7%)			308 – (100%)		
**Feeling**	79	64	3	117	71	0	112	56	0	22	33	0	330	224	3
**A3 SUM**	102 – (45.5%)			67 – (29.9%)			45 – (20.0%)			10 – (4.4%)			224 – (100%)		
**Feeling**	50	50	2	80	54	0	76	59	0	23	24	0	229	187	2
**A4 SUM**	108 – (50%)			73 – (33.7%)			32 – (14.8%)			3 – (1.3%)			216 – (100%)		
**Feeling**	60	47	1	78	65	1	60	36	0	13	4	0	211	152	2
**A5 SUM**	92 – (52.8%)			53 – (30.4%)			25 – (14.3%)			4 – (2.9%)			174 – (100%)		
**Feeling**	48	42	2	66	39	1	41	33	1	0	21	0	155	135	4
**A6 SUM**	100 – (47.6)			73 – (34.7%)			32 – (15.2%)			5 – (2.3%)			210 – (100%)		
**Feeling**	53	45	2	85	58	3	62	33	1	14	7	0	214	143	6
**A7 SUM**	75 – (52.4%)			43 – (30.0%)			21 – (14.6%)			4 – (2.7%)			143 – (100%)		
**Feeling**	44	28	3	56	30	0	29	34	0	8	8	0	137	100	3
**A8 SUM**	101 – (54.0%)			55 – (29.4%)			28 – (14.9%)			3 – (1.6%)			187 – (100%)		
**Feeling**	50	50	1	62	48	0	42	42	0	12	0	0	166	140	1
**A9 SUM**	192 – (60.1%)			91 – (28.5%)			27 – (8.4%)			9 – (2.8%)			319 – (100%)		
**Feeling**	112	75	5	128	54	0	57	24	0	21	22	0	318	175	5
**N1 SUM**	129 – (53.9%)			58 – (24.2%)			40 – (16.7%)			12 – (5.0%)			239 – (100%)		
**Feeling**	40	89	0	38	78	0	50	70	0	27	34	0	155	271	0
**N2 SUM**	10 – (40%)			6 – (24%)			7 – (28%)			2 – (8.0%)			25 – (100%)		
**Feeling**	4	6	0	3	3	0	8	9	1	3	5	0	18	23	1
**N3 SUM**	11 – (33.3%)			14 – (42.4%)			6 – (18.1%)			2 – (6.0%)			33 – (100%)		
**Feeling**	4	7	0	15	13	0	8	10	0	5	4	0	32	34	0
**N4 SUM**	4 – (40%)			5 – (50%)			1 – (10%)			0 – (0.0%)			10 – (100%)		
**Feeling**	1	3	0	5	5	0	3	0	0	0	0	0	9	8	0
**N5 SUM**	23 – (56.0%)			12 – (29.2%			4 – (9.7%)			2 – (4.8%)			41 – (100%)		
**Feeling**	9	14	0	8	16	0	6	6	0	4	5	0	27	41	0

**Index for** Table 3.

NTG - Nitroglycerin.

(+) – feeling better,

(−) – feeling worse,

(o) - feeling the same

**Fig. 1:**
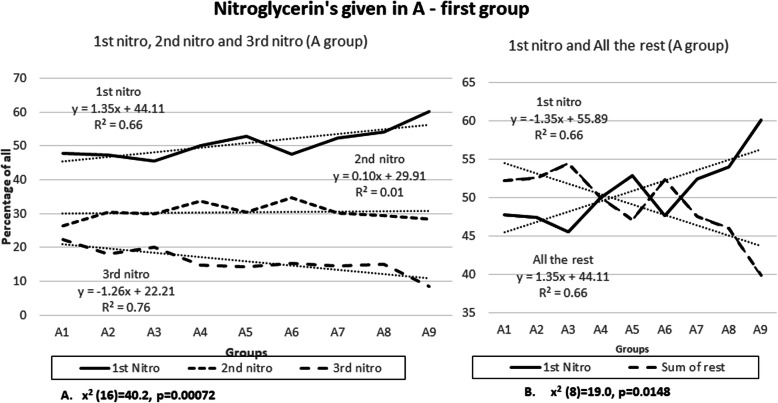
Nitroglycerin’s given A-first group

Giving the 1st dose of nitroglycerin a few minutes after aspirin resulted in a reduction of patients requiring a 3rd dose of nitroglycerin (Fig. 1a). The 1 dose nitroglycerin group has a linear upward trend line progression from A1 to A9 groups, with a slope of 1.35 (R^2^ 0.66) and the 3 dose nitroglycerine group has downward slope of −1.26 (R^2^ 0.75). This results in a 23.5% reduction of a 3rd nitroglycerin dose in the A9 group compared to the A1 group. Chi square of independence was found to be significant (×2 (16) =40.21, *p* = 0.00072). This finding has a linear progression from the A1 to the A9 group.

The same correlation was found when the 1 dose nitroglycerine group is compared to the sum of all additional nitroglycerin given (Fig. 1b). The analysis showed that there is a 24.3% reduction in dosing of any additional nitroglycerin after the first one in the A9 group with significant linear progression across all groups (R^2^ 0.66). It is significant that in the A1 group there were 11.74% more patients who received additional nitroglycerin compared to the A9 group where 12.56% of patients received only 1 dose of nitroglycerin compared to all additional nitroglycerin. These data have significant chi square of independence (× 2 (8) =19.0, *p* = 0.0148).

This correlation was not found within the N1 group because the data were analyzed and were found statistically insignificant using chi square of independence.

**Fig. 2:**
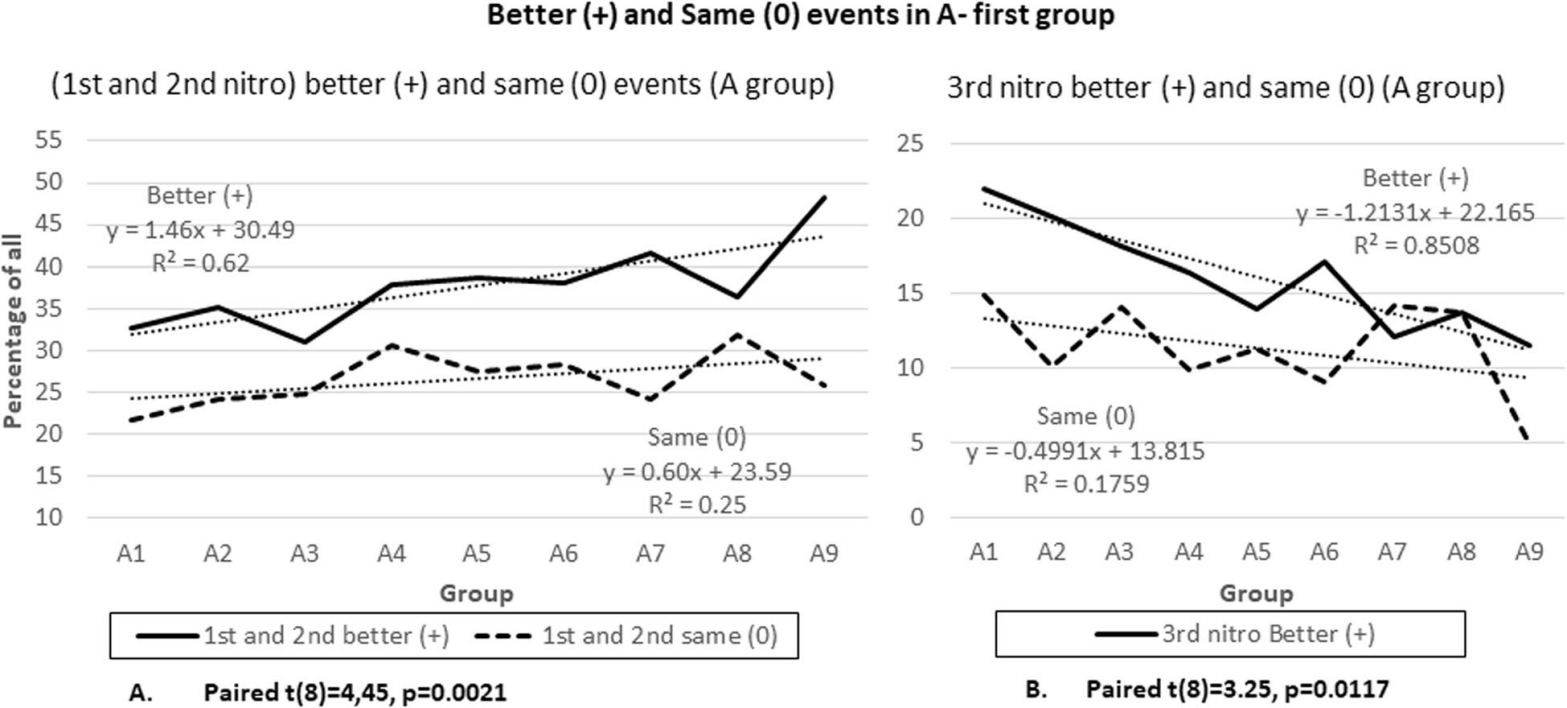
Better (+) and Same (0) events a A-first group

The second set of analysis analyzed the subjective feeling of the patients after receiving each dose of medication. Similarly, as in the first set of analysis there is a linear trend from A1 to A9 groups. More patients were feeling “better” (+) than “same” (0) in groups where they received the first dose of nitroglycerin a few minutes after aspirin. Figure 2a shows the sum of 1 nitro and 2 nitro groups. The better (+) event has an up-sloping progression with a slope of 1.46 (R^2^ of 0.62) and the “same” or (0) event has a trend line slope of 0.6 (R^2^ 0.25). This means that the trend lines are diverging and this results in 7.7% more (+) events than (0) in A9 group compared to (A1) group. Figure 2a was tested with a paired t-test because it is a set of paired data, derived from 1 and 2 nitroglycerine groups. There was a significant difference in the scores for “better” (+) (M = 160.56, SD = 62,52) and “same” (0) events (M = 111.56, SD = 35,74), conditions: t (8) =4,45, *p* = 0.0021.

Even though both 1 and 2 nitroglycerine groups independently had a similar linear progression and divergence they are presented together for simplicity sake. Same was done for 3rd and all the rest groups.

The 3rd and the rest of nitroglycerine subgroups had the opposite trend and the trend lines converge (Fig. 2b). This is shown in Fig. 2b where the “better” or (+) events have downward slope of − 1.37 (R^2^ 0.66) and the “same” or (0) events have slope of − 0.72 (R^2^ 0.27). This results in a change of negative 5.85% from A1 to A9, or more patients reported “same” than “better”. Data validity was also tested with paired t test with significant differences in the scores for “better” (+) (M = 91,67 SD = 60,8) and “same” (0) events (M = 64,67, SD = 35.79) conditions: t (8) =2.73, *p* = 0.0258.

This correlation was not found within the N1 group because the data were analyzed and were found insignificant using paired t-test.

**Fig. 3:**
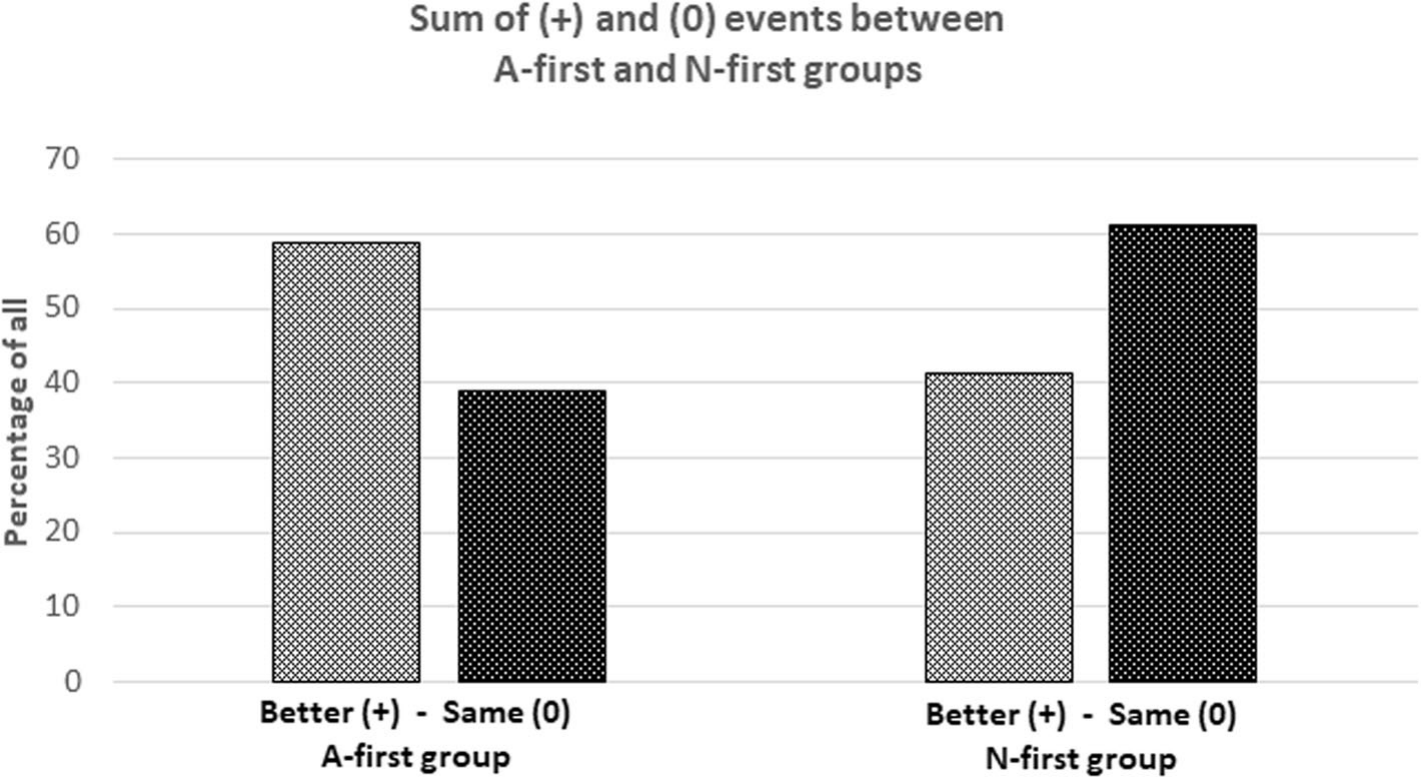
Sum of (+) and (0) event between A-first and N-first group

Figure 3 presents the sum of all “better” (+) and “same” (0) events for A and N groups. There is a 39.6% difference between “better” and “same” events between A-group and N-group. Standalone statistical analysis of N-group was not possible because the data were not significant. However, even though statistical analysis of N-group was not significant it is a large number of patients (348). Because of this, the comparison of the sum of both groups has significance of its own showing the complete reverse status of these two groups.

In order to exclude that this trend of reduced nitroglycerin administration in later groups is due to increased use of pain medications we also tested administration of pain medications. We measured only pain medications given after the first nitroglycerin in order to better correlate between them. Patients received 3 types of pain medications:

- Morphine - 497 given
- Fentanyl - 302 given
- Midazolam - 7 given.

Descriptive data of this analysis are presented in Table 4.

**Table 4:** Descriptive data for opioids given

	Pts. that received opioids	Total drugs given	Total No. of patients
**A1**	82	137	465
**A2**	70	109	308
**A3**	42	60	224
**A4**	32	44	216
**A5**	32	53	174
**A6**	35	56	210
**A7**	32	57	143
**A8**	30	50	187
**A9**	56	78	319
**N1**	41	62	234
**N2**	6	12	26
**N3**	5	7	25
**N4**	1	1	10
**N5**	16	23	53

**Fig. 4:**
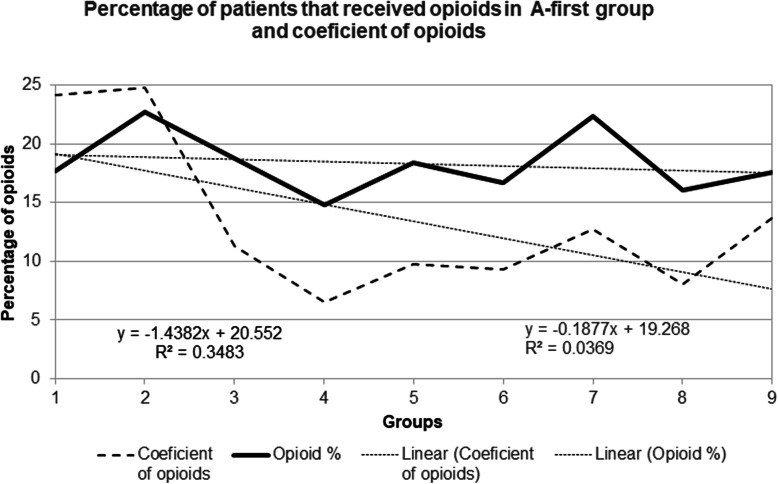
Percentage of patients that received opioids in A-first group and coefficient of opioids

Graphic presentation in opioids given is presented in Fig. 4. It is evident that the total number of patients that received opioids has a very weak downward slope of − 0.18 (with R^2^ 0.036) from A1 to A9. However, we designed a coefficient of opioids to include the number of opioids given per group. The need to give opioids is directly proportional to the number of patients that received opioids and the number of opioids that they received and is inversely proportional by the number of patients that are in the group. This can mathematically be presented as coefficient:

### Coefficient of opioids = (pts. that received opioids x opioids given) / Total number of patients

This coefficient of opioids regards the number of patients that received opioids and the number of opioids they received. This coefficient has a significant downward trend line with slope of 1.44 with a relatively significant R^2^ of 0.34. This shows that in later groups a smaller percentage of opioid “load” was needed. However, these data have an insignificant chi square of independence (× 2 (16) =18.1, *p* = 0.316). This means that giving opioids is not dependent on the previous nitroglycerine given or has any relationship between the groups. In other words, giving opioids did not influence the administration of nitroglycerin in the patients. (See supplementary 10).

N-first group had an insignificant number of opioids given and is not presented.

In order to find some reason why some patients received nitroglycerin soon after aspirin and some later (A1 through A9), or why some patients received nitroglycerin first we analyzed a number of vital signs and demographic data recorded in the NEMSIS dataset (See Supplementary 4, 5). We tested the averages of:

- Systolic blood pressure (SBP),
- Heart rate,
- Patient acuity at start and disposition
- Pulse oximetry findings,
- Pain severity on a scale from 1 to 10,
- Type of ischemia (STEMI or NON-STEMI),
- Age,
- Gender differences.
- **Type of ECG changes**

Descriptive data for these analyses are presented in Table 5.

**Table 5:** Descriptive data for vital signs and demographic data

**A-FIRST GROUP**	**A1**	**A2**	**A3**	**A4**	**A5**	**A6**	**A7**	**A8**	**A9**
2003SBP (mmHg)	147.01	144.57	141.65	148.57	143.87	141.13	140.02	136.69	140.75
Heart rate	86.16	84.55	84.39	83.25	84.65	83.9	87.17	83.4	82.08
**Acuity**									
Red 1st	41.51	43.26	53.72	47.11	43.16	49.59	50.68	46.53	34.95
Yellow 1st	47.92	48.31	34.71	45.45	45.26	40.50	45.21	42.57	50.00
Green 1st	10.57	8.43	11.57	7.44	11.58	9.92	4.11	10.89	15.05
Red 2nd	27.17	36.52	39.67	33.88	32.63	39.67	21.92	31.68	27.96
Yellow2nd	46.79	40.45	33.88	42.15	35.79	37.19	52.05	46.53	44.62
Green 2nd	26.04	23.03	26.45	23.97	31.58	23.14	26.03	21.78	27.42
SpO2	96.02	96.79	96.14	96.86	96.15	96.25	95.96	95.78	96.66
Respirations	18.09	18.78	19.13	18.84	20.44	19.13	19.27	18.9	18.58
Pain scale	6.08	6.56	6.55	6.01	6.08	6.08	6.12	6.47	5.94
STEMI	431	286	211	198	161	196	134	173	298
NON-STEMI	33	22	13	18	13	14	9	14	28
% STEMI	92.89	92.86	94.2	91.67	92.53	93.33	93.71	92.51	91.41
% NON-STEMI	7.11	7.14	5.8	8.33	7.47	6.67	6.29	7.49	8.59
MALES	329	213	157	144	121	152	91	135	209
FEMALES	135	94	66	72	53	58	52	50	110
AGE	60.88	61.52	63.68	63.23	62.76	62.78	62.9	64.77	62.87
%MALE	70.91	69.38	70.4	66.67	69.54	72.38	63.64	72.97	65.52
%FEMALE	29.09	30.62	29.6	33.33	30.46	27.62	36.36	27.03	34.48
RATIO M/F	2.44	2.27	2.38	2	2.28	2.62	1.75	2.7	1.9
**N-FIRST GROUP**	N1	N2	N3	N4	N5				
SBP (mmHg)	144.03	142.4	130.95	132.6	149.19				
Heart rate	85.37	85.9	93	79	96				
SpO2	96.56	93.25	94.79	87	94.94				
Respirations	19.45	25.03	18.64	17.8	21.14				
Pain scale	6.19	4.69	5.16	4.44	6.25				
STEMI	214	26	24	9	50				
NON-STEMI	20	0	1	1	3				
% STEMI	91.45	100	96	90	94.34				
% NON-STEMI	8.55	0	4	10	5.66				
MALES	163	18	18	8	31				
FEMALES	70	8	7	2	22				
AGE	65.06	66.76	68.08	59.8	67.05				
%MALE	69.96	69.23	72	80	58.49				
%FEMALE	30.04	30.77	28	20	41.51				
RATIO M/F	2.33	2.25	2.57	4	1.41				

**Index for** Table 5**:**

SBP (mmHg) – average systolic blood pressure in mmHg

Heart rate – average heart rate

Acuity - at first situation (1st) and at dispossition (2nd). Red is worst acuity, Green is best acuity

SpO2 – average SpO2

Respirations – average respirations per minute

Pain scale – average pain on a scale from 1 to 10

STEMI – total number of patients with STEMI

NON-STEMI – total number of patients with NON-STEMI

% STEMI – percentage of STEMI patients

% NON-STEMI – percentage of NON-STEMI patients

MALES – total number of males

FEMALES – total number of females

AGE – average age of patents

%MALE – percentage of males

%FEMALE – percentage of females

RATIO M/F – ratio between males and females

**Fig. 5:**
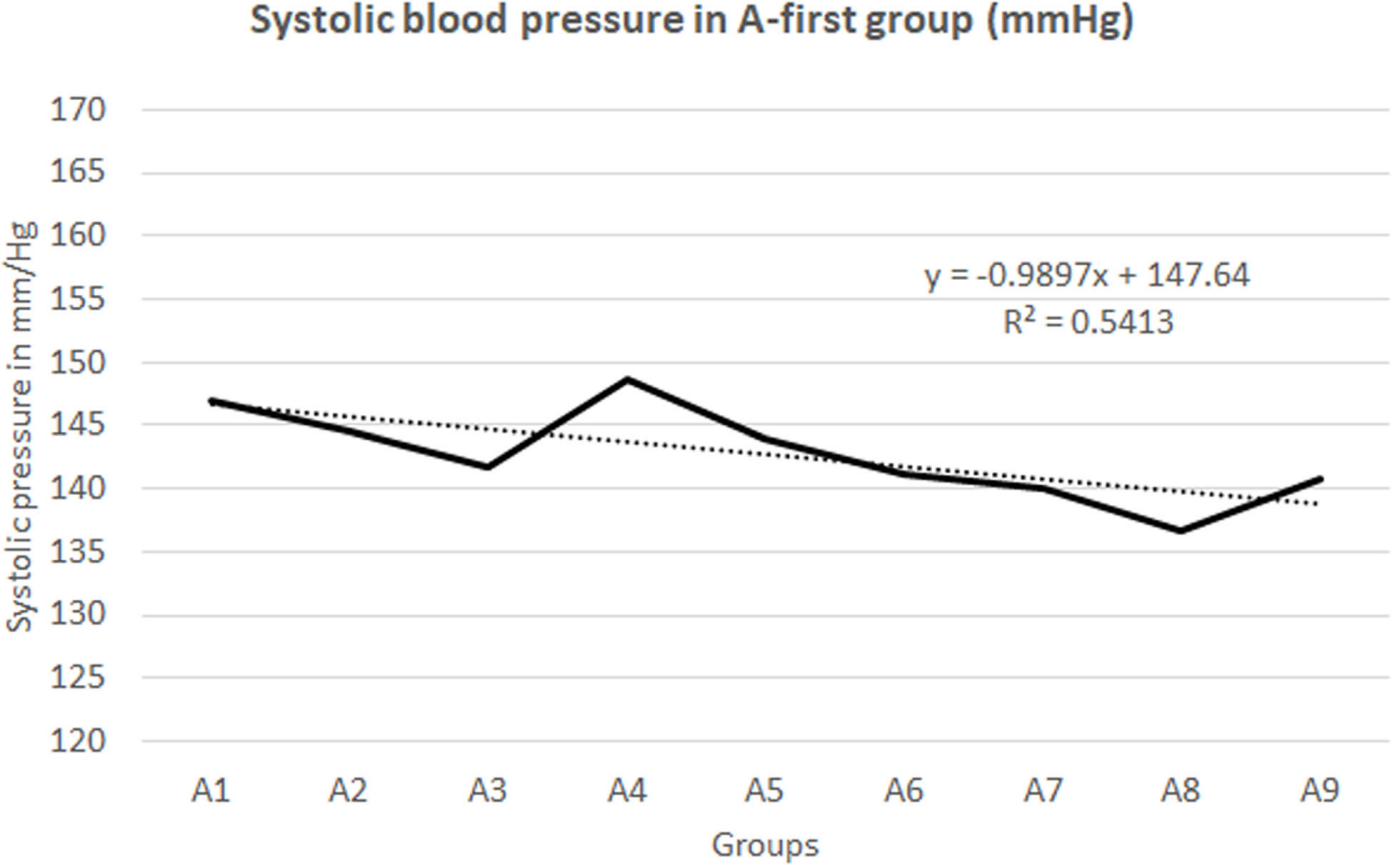
Systolic blood pressure in A-first group (mmHg)

**Fig. 6:**
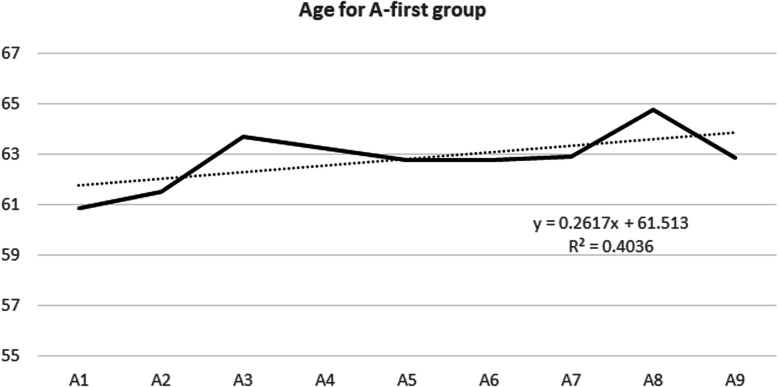
Age for A-first group

No significant correlation was found for any of the vital signs, except for systolic blood pressure and age of the patients as presented on Figs. 5 and 6. Patient acuity test showed improved acuity at disposition for patients marked Red and Yellow but data were found statistically insignificant. See supplementary 16 and 17.

Figure 5 shows a down sloping trend line with slope of 0.99 (R^2^ of 0.54) which shows that on average A9 group had 8.9 mmHg lower systolic blood pressure compared to A1 group, and this is a consistent trend from A1 to A9 group. To some patients, systolic blood pressure was measured several times and we only compared the average for the whole group. The rationale behind this decision was to find a pattern that can explain the postponing of nitroglycerin administration.

We found another significant trend in the A-first group regarding the average age. There is an up-sloping trend (slope 0.26 with R^2^ of 0.4) for the age of the patients from A1 to A9 group as presented in Fig. 6. This means older patients received nitroglycerin later in A-group.

No similar correlations and significant trends were found in N-group.

**Fig. 7:**
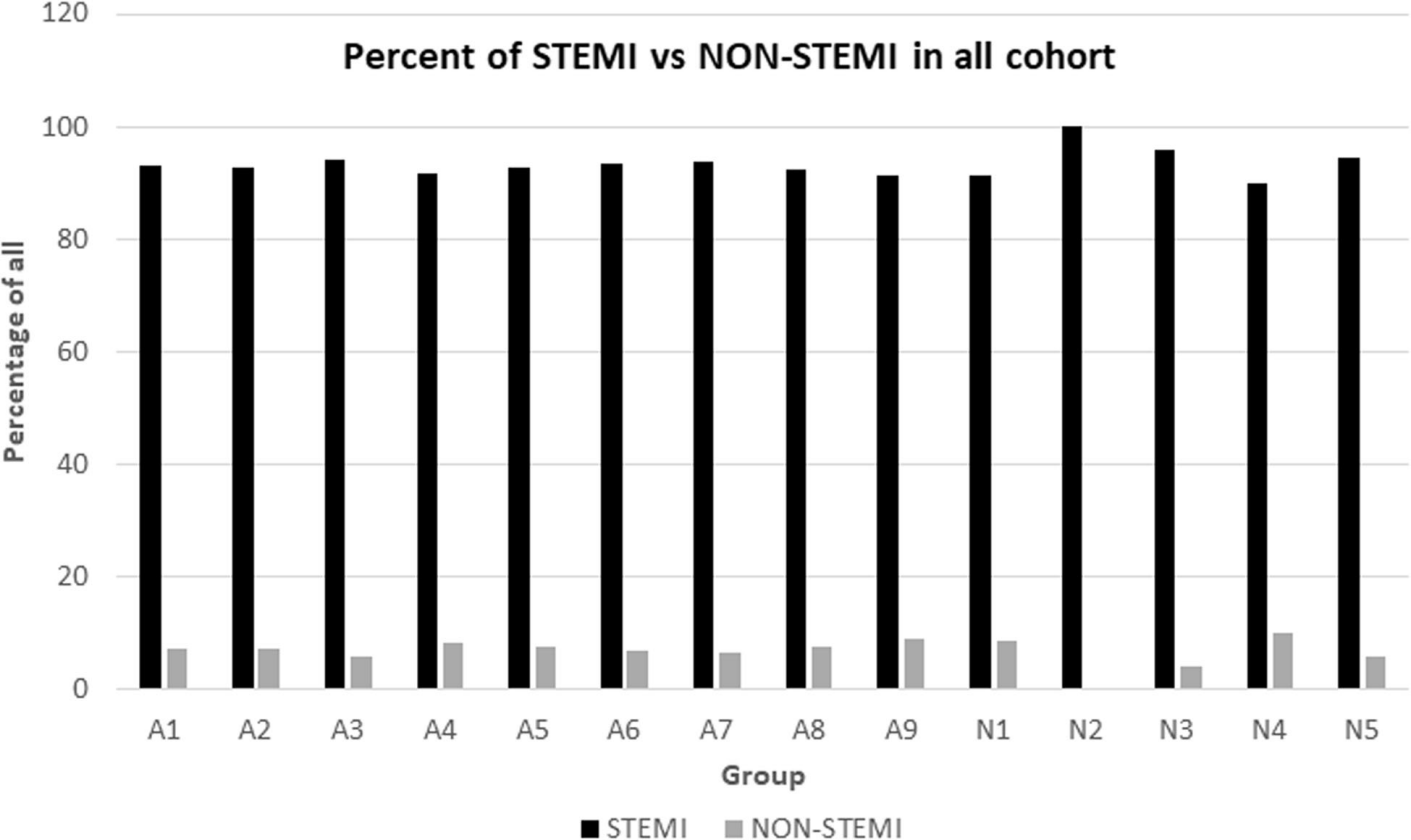
Percent with STEMI vs NON-STEMI in all patients 9 both groups

We analyzed which type of ECG changes were most prevalent within the cohort, and the result showed that than 92.71% were STEMI myocardial ischemia, and 7.28% were NON_STEMI as shown in Fig. 7. (See Supplementary 12, 13, 14) N-first group alone had similar ratio. Out of 348 patients in N-first group 323 cases or 92.81% were STEMI and 25 or 7.19% were NON-STEMI patient. (See supplementary 15).

Finding that more that 92.71% of all cohort-patients were with STEMI changes was surprising and we wanted to analyze if there are any changes in the findings in this subgroup. The same sets of analyses were conducted on the exclusive 2404 STEMI patients. Again only A-First group is presented, N-First group had insufficient data.

**Fig. 8:**
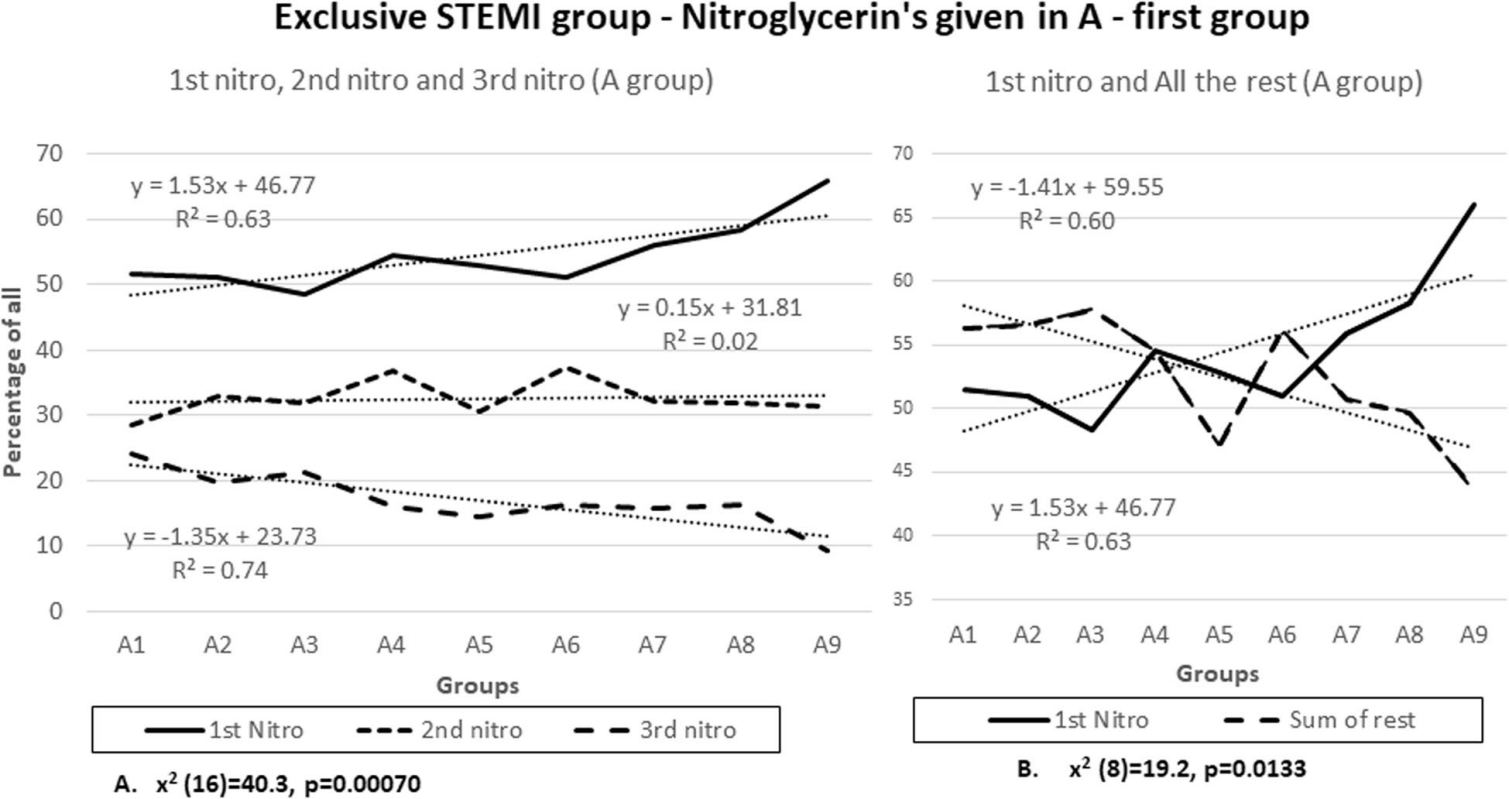
Exclusive STEMI group – Nitroglycerin’s given in A-first group

Figure 8a is very similar to Fig. 1a. The 1 dose nitroglycerin group has a linear upward trend line progression from A1 to A9 groups, with a slope of 1.53 (R^2^ 0.63) and the 3 dose nitroglycerine group has downward slope of − 1.35 (R^2^ 0.74). This results in a 25.9% (improvement of 2.4%) reduction of a 3rd nitroglycerin dose in the A9 group compared to the A1 group. Chi square of independence was found to be significant (× 2 (16) = 40.3, *p* = 0.00070).

The same correlation was found when the 1 dose nitroglycerine group is compared to the sum of all additional nitroglycerin given (Fig. 8b). The analysis showed that there is a 26.46% (improvement of 2.16%) reduction in dosing of any additional nitroglycerin after the first one in the A9 group with significant linear progression across all groups (R^2^ 0.63 and 0.60). These data have significant chi square of independence (× 2 (8) = 19.2, *p* = 0.0133).

**Fig. 9:**
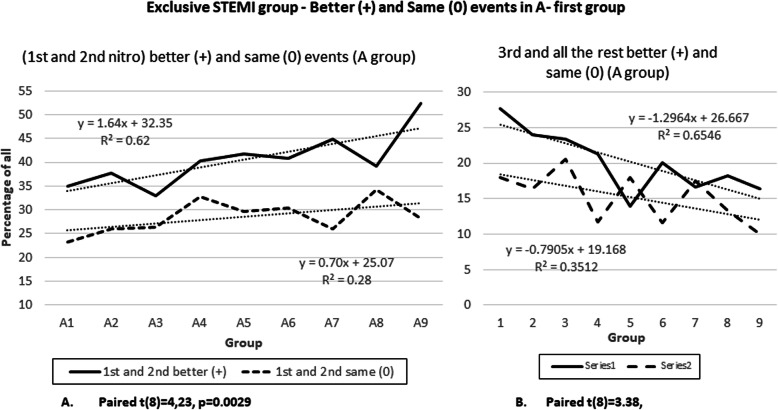
Exclusive STEMI group – Better (+) and Same (0) events in a A-first group

The second set of analysis shown in Fig. 9 is also very similar to Fig. 2. The better (+) event has an up-sloping progression with a slope of 1.64 (R^2^ of 0.62) and the “same” or (0) event has a trend line slope of 0.7 (R^2^ 0.28). This means that the trend lines are diverging and this results in 8.46% (improvement of 0.76%) more (+) events than (0) in A9 group compared to A1 group. The paired t-test showed a significant difference in the scores for “better” (+) (M = 146.56, SD = 55,96) and “same” (0) events (M = 104.00, SD = 33,49), conditions: t (8) = 4,23, *p* = 0.0029.

The 3rd and all remaining nitroglycerine subgroups again had the opposite trend and the trend lines converge (Fig. 2b). This is shown in Fig. 2b where the “better” or (+) events have downward slope of − 1.29 (R^2^ 0.65) and the “same” or (0) events have slope of − 0.79 (R^2^ 0.35). This results in a change of negative 4.55% (reduced by 1.3%) from A1 to A9, or more patients reported “same” than “better”. Data validity was also tested with paired t test with significant differences in the scores for “better” (+) (M = 86,67 SD = 56,99) and “same” (0) events (M = 62,22, SD = 35.34) conditions: t (8) = 2.83, *p* = 0.0221.

Both test in the exclusive STEMI group of 2404 patients showed small improvements over the overall 2594 patients included in the study. The improvements are presented in Table 6.

**Table:** Table 6

	Improvenents in exclusive STEMI group (2404 patients) over the whole cohort of 2594 patients	
1	- Figure 1a improved by 2.4%	meaning less 3rd nitroglycerin’s were given in A9 compared to A1 (compared to whole cohort analysis)
2	Figure 1b improved by 2.16%	meaning more patients received only 1 nitroglycerin in group A9 compared to A1 (job was done with 1 nitroglycerin). (compared to whole cohort analysis)
3	Figure 2a improved by 0.76%	meaning more patients were feeling better (+) than same (0) in A9 compared to A1 in the combined 1st and 2nd nitroglycerin group. (compared to whole cohort analysis)
4	Figure 2b was reduced by 1.3%	meaning less patients were felling same (0), or more patients reported better (+) in A9 compared to A1 in the combined 3rd and the rest group. (compared to whole cohort analysis)

## Discussion

The results show that during acute coronary syndrome (mostly STEMI) it is beneficial to give aspirin first and give nitroglycerin several minutes later. Trend lines in Figs. 1 and 2 show that this “intervention” is associated with more than 20% reduced need for a third nitroglycerin dose and a more than 20% reduced need of any additional nitroglycerin if the first nitroglycerin is given 10 min after aspirin (10 min is selected as a cut-off from the trend line as a round number). This intervention is additionally associated with reduced need for opioids. In addition, with this “intervention” patients are feeling more than 7% better after administering the first or the second nitroglycerin when nitroglycerin was given 10 min after aspirin. In general, patients in A-group were feeling 39.6% better compared to N-group (Fig. 3).

Additionally, exclusive STEMI group (2404 patients or 92.71%) produced almost exact trends. Table 6 shows that there were small improvements in all aspects compared to the whole group of 2954 patients. This is important because ECG with STEMI changes is considered a definite sign of acute coronary syndrome, and the intervention is apparently slightly more effective in this group.

The results are linear and time-dependent (giving nitroglycerin later has a larger benefit), nevertheless, the exact timing when nitroglycerin should be given cannot be answered by this study.

Giving opioids has a down sloping trend line from A1 to A9 groups, but this data was found to be statistically insignificant (Fig. 4), The coefficient of opioids has a significant down-sloping trend and may indicate reduced opioids administration. The conclusion, however, is that in later groups nitroglycerin was not replaced with increased opioid use. The most logical answer why these patients received less nitroglycerin is that they did not had any need to receive any additional nitroglycerin because they were feeling better.

Reduced initial systolic blood pressure may be the reason why some patients received their first dose of nitroglycerin later compared to others (Fig. 5). Nitroglycerin can cause a dramatic drop in systolic blood pressure and some medical professionals may have been hesitant in administering the nitroglycerin sooner. This may be the reason why medical personnel mostly give nitroglycerin in patients with STEMI ischemia. Majority, or 92.71% of the patients in the dataset, who had received nitroglycerin had STEMI ischemia (Fig. 7).

The age of the patient may also have played a role in deciding to give the nitroglycerin later in some patients because the medical personnel may have been more cautious with patients who were older and had lower systolic blood pressures (Fig. 6).

The data show that giving nitroglycerin before aspirin may lead to worse subjective feeling of the patient as presented in Fig. 3.

As mentioned, Gawaz [6] shows that during acute coronary syndrome the thrombus does not always lead to complete occlusion of the blood vessel, and in some cases the blood circulation breaks the thrombus in essence, an event of revascularization. Giving aspirin several minutes before nitroglycerin may potentiate this process, and this may be the reason why patients who received their first nitroglycerin after aspirin were feeling better.

The reason for this may be found in the qualities and nature of these two drugs.

Sean et al. [10] found that chewed aspirin reaches maximum blood concentration after 27 min. Mark and Byron [11] however, in a randomized and experimental study show that 5 min after chewing aspirin the level of thromboxane B2 (TxB2) in the serum decreased 50% from the normal level. They claim this concentration of aspirin is enough for gaining the anti-aggregation effect of the aspirin.

Aspirin affects the thrombus in a qualitative and quantitative way. Normally the thrombus has a compact structure with small pores and thin fibrin fibers and it is associated with more severe forms of coronary artery disease [12, 13]. Aspirin affects the structure during the formation of the thrombus making it softer and more easier to dissolve [14, 15]. Aspirin also affects the structure of already formed thrombus, forming thicker fibrin fibers with bigger pores which causes reduced stability of the thrombus and a greater ability to dissolve [14]. In in-vitro conditions aspirin reduces the speed of formation of the thrombus, acts on its structure and size, i.e. creates smaller thrombus [11, 16].

Nitroglycerin is given in acute coronary syndrome for its vasodilatory effect. Munemasa et al. [17] showed significant increases in the diameter of the coronary blood vessels before and after the application of nitroglycerin with variation ranged from 7.54 to 22.26%, whereas smaller blood vessels showed vasodilatory effect of 16.91% compared to the bigger blood vessels which showed 11.35%. Tadamura et al. [18] showed that this vasodilatory effect is most evident exactly at the area where the viable tissue with ischemia is, i.e. where it is needed the most.

Because of these “qualities” of aspirin and nitroglycerin, if aspirin is chewed and present in the circulation a short period of time before nitroglycerin (5 to 10 min), it may manifest its anti-aggregatory effect. When nitroglycerin is administered second and causes local vasodilation, that may prevent the propagation of the thrombus and cause re-occlusion of the blood vessel. This may be the mechanism causing the results in this study.

It is important to note at this time that this “intervention” can be viewed as a “principle of work”. It can be done with other anti-aggregatory and vasodilatory drugs such as ticagrelor and nitroglycerin. ESC guidelines for 2017 recommend ticagrelor as a first choice option in patients with ACS, with or without ST elevation and regardless of further treatment strategy [19]. One can speculate that the “intervention” may also improve the efficacy of ticagrelor.

It must be noted that there are some aspects of the study that may lead to bias or alternate interpretation.

In Fig. 2b we see that patients are actually feeling worse in the A9 group. This however, is in concordance with the hypothesis because the 3rd and the rest dose nitroglycerine group probably consists only of resistant patients, where there is a need for more invasive procedures. This actually only confirms the validity of the data because it is consistent with the first set of analysis where the 3rd dose nitroglycerine group has a downward slope. In other words, in the A1 group only after the 3rd nitroglycerin dose were the patients feeling better, and in A9 more patients were treated with only 1 nitroglycerin and only the most resistant needed a 3rd dose. This is clearly seen in Fig. 1b.

Another concern is that some patients did not receive additional nitroglycerin because they were feeling worse after the first one and medical personnel just did not give another one. The analysis of the vital signs and demographic data did not show that this is the case. Also, the same trends for both sets of analysis (number of nitroglycerin doses and subjective feeling of the patient) and reduced opioid load in later groups should minimize this bias.

Could N-First group consist of solely STEMI patients with more severe condition? We analyzed the ratio of STEMI-NON-STEMI in N-first group alone and found similar ratios as whole cohort (92.8% STEMI and 7.19% NON-STEMI). Thus means N-First group is no different from the whole cohort. Simply, some emergency personnel decided to give nitroglycerin first in those patients.

Another possible bias is that NON_STEMI group (7.28% of all) may contain some patients that did not have acute coronary syndrome (NON-STEMI ECG changes and chest pain may have been caused by other problems). Acute coronary syndrome is however highly suggestive in majority of this group. Some of the NON-STEMI patients were tested for troponin levels on site, because some providers use point of care troponin testing (we don’t know the exact percentage though). The ECG findings, chest pain, the medication that was given and the judgement of the emergency personnel to treat it and report it as such should minimize this bias. The solution of this problem is the set of analyses on the exclusive STEMI patients. This analysis produced basically the same results and trend-lines but showed 0.7 to 2.4% improvements in all 4 aspects of the analysis. This analysis shows that even if this was a case these patients represent a very small or non-existent percentage in NON-STEMI group. More importantly, again confirms the efficacy of the intervention in the patients with acute coronary syndrome.

A concern is the consistency of data gathering for this study. Events were recorded in real time and we cannot be sure that they have been entered correctly in the rush of the situation. Also, maybe in the heat of the moment a wrong code was pressed or patients ECG was misinterpreted as ACS. For this however, NEMSIS database has a system of validation and data cleaning process. As mentioned above, all data are checked for completeness, logical consistency and proper formatting. Any data that does not pass this process are rejected and not included in the database [9]. This means that the paramedics cannot completely neglect the procedure and we believe that this is not frequent enough that would affect the results.

Regardless of the above concerns we believe that the fact that 4 sets of analyses (exclusive STEMI group included) resulted in similar results is a confirmation of the validity of the data. Further research is necessary in order to prove or disprove the findings of this study.

## Conclusion

This study found an association between giving aspirin before nitroglycerin in patients with STEMI ECG changes and a reduction in subjective pain. The result was time-dependent, in that giving nitroglycerin a few minutes after aspirin was associated with reduced additional doses of nitroglycerine or opioids. Future prospective trials are needed to prove or disprove the findings of this retrospective study.

### Conflict of interest statement

There was no conflict of interest in this study neither personal, professional, or financial. The author has full access to all data for the study and all supplementary materials.

## Supplementary Information

**Additional file 1.**

**Additional file 2.**

**Additional file 3.**

**Additional file 4.**

**Additional file 5.**

**Additional file 6.**

**Additional file 7.**

**Additional file 8.**

**Additional file 9.**

**Additional file 10.**

**Additional file 11.**

**Additional file 12.**

**Additional file 13.**

**Additional file 14.**

**Additional file 15.**

**Additional file 16.**

**Additional file 17.**

## Data Availability

All data are available as supplementary material provided with this manuscript. The NEMSIS dataset is available online.
