# Supplementary material for: The timing of administering aspirin and nitroglycerin in patients with STEMI ECG changes alter patient outcome
Source: BMC Emerg Med. 2021 Nov 17;21:137. doi: 10.1186/s12873-021-00523-2 (PMC8597308; doi:10.1186/s12873-021-00523-2)
Supplement: Supplementary file 9 — Additional file 9. [file 12873_2021_523_MOESM9_ESM.doc]

**PYTHON SOFTWARE 1.**

import sys

target_diagnosis = sys.argv[2:]

for line in sys.stdin:

tokens = line.strip().split("~|~")

if tokens[0] == "'PcrVitalECGGroupKey'":

sys.stdout.write(line)

else:

eVitals_03 = tokens[2].strip()

if eVitals_03 in target_diagnosis:

sys.stdout.write(line)

**PYTHON SOFTWARE 2.**

import sys

vital_keys = set()

vital_keys_in = open(sys.argv[1], "rt")

# read all vital keys from PCRVITALECGGROUP_filtered.txt

for line in vital_keys_in:

tokens = line.strip().split("~|~")

if tokens[0] != "'PcrVitalECGGroupKey'":

vital_key = tokens[1].strip()

vital_keys.add(vital_key)

# sys.stdout.write(repr(vital_keys))

# filter only those lines from FACTPCRVITAL.txt with the desired vital keys

for line in sys.stdin: # stdin is FACTPCRVITAL.txt

tokens = line.strip().split("~|~")

if tokens[0] == "'PcrVitalKey'":

sys.stdout.write(line)

else:

vital_key = tokens[0].strip()

if vital_key in vital_keys:

sys.stdout.write(line)

**PYTHON SOFTWARE 3**

import sys

prc_keys = set()

prc_keys_in = open(sys.argv[1], "rt")

# read all prc keys from FACTPCRVITAL_filtered.txt

for line in prc_keys_in:

tokens = line.strip().split("~|~")

if tokens[0] != "'PcrVitalKey'":

prc_key = tokens[1].strip()

prc_keys.add(prc_key)

# sys.stdout.write(repr(prc_keys))

# filter only those lines from FACTPCRMEDICATION.txt with the desired prc keys

for line in sys.stdin: # stdin is FACTPCRMEDICATION.txt

tokens = line.strip().split("~|~")

if tokens[0] == "'PcrMedicationKey'":

sys.stdout.write(line)

else:

prc_key = tokens[1].strip()

if prc_key in prc_keys:

sys.stdout.write(line)

**PYTHON SOFTWARE 4**

import sys

import time

invalid_results = set()

invalid_results.update(["7701001", "7701003", "7701005"])

filtered_medicines = sys.argv[1:]

improvement_signs = {"9916001": "+", "9916003": "0", "9916005": "-"}

prev_patient_id = 0

patient_id = 0

patient_lines = []

patient_medicines = []

# get aspirin then nitro number

def get_an_number(duration):

duration /= 60.0

if duration <= 1.0:

return "1"

elif duration <= 2.0:

return "2"

elif duration <= 3.0:

return "3"

elif duration <= 4.0:

return "4"

elif duration <= 5.0:

return "5"

elif duration <= 7.0:

return "6"

elif duration <= 9.0:

return "7"

elif duration <= 12.0:

return "8"

else:

return "9"

# get nitro then aspirin number

def get_na_number(duration):

duration /= 60.0

if duration <= 2.0:

return "1"

elif duration <= 4.0:

return "2"

elif duration <= 6.0:

return "3"

elif duration <= 8.0:

return "4"

else:

return "5"

# is aspirin then nitro group

def is_an_group(patient_lines):

for line in patient_lines:

tokens = line.strip().split("~|~")

if tokens[3].strip() == "Aspirin":

return True

elif tokens[3].strip() == "Nitroglycerin":

return False

return False

# is nitro then aspirin

def is_na_group(patient_lines):

for line in patient_lines:

tokens = line.strip().split("~|~")

if tokens[3].strip() == "Nitroglycerin":

return True

elif tokens[3].strip() == "Aspirin":

return False

return False

def are_all_results_valid(patient_lines):

for line in patient_lines:

tokens = line.strip().split("~|~")

if tokens[-4].strip() in invalid_results:

return False

return True

def are_all_times_valid(patient_lines):

for line in patient_lines:

tokens = line.strip().split("~|~")

try:

time_point = time.strptime(tokens[-1].strip(), "%Y-%m-%d %H:%M:%S")

except ValueError:

return False

return True

def get_first_aspirin_index(patient_lines):

for i in range(0, len(patient_lines)):

line = patient_lines[i]

tokens = line.strip().split("~|~")

if tokens[3].strip() == "Aspirin":

return i

return None # should never be reached

def get_first_nitro_index(patient_lines):

for i in range(0, len(patient_lines)):

line = patient_lines[i]

tokens = line.strip().split("~|~")

if tokens[3].strip() == "Nitroglycerin":

return i

return None # should never be reached

def add_aspirin_labels(patient_lines):

i0 = get_first_aspirin_index(patient_lines)

tokens = patient_lines[i0].strip().split("~|~")

time0 = time.strptime(tokens[-1].strip(), "%Y-%m-%d %H:%M:%S")

t0 = time.mktime(time0)

assessment = " ~|~ A"

counter = 0

for i in range(i0 + 1, len(patient_lines)):

line = patient_lines[i]

tokens = line.strip().split("~|~")

if tokens[3].strip() == "Nitroglycerin":

counter += 1

if counter == 1:

time1 = time.strptime(tokens[-1].strip(), "%Y-%m-%d %H:%M:%S")

t1 = time.mktime(time1)

num = get_an_number(t1 - t0)

else:

num = str(counter)

improvement = tokens[-4].strip()

assessment = assessment + num + improvement_signs[improvement]

patient_lines[i] = line.strip() + assessment + "\n"

def add_nitro_labels(patient_lines):

i0 = get_first_nitro_index(patient_lines)

tokens = patient_lines[i0].strip().split("~|~")

time0 = time.strptime(tokens[-1].strip(), "%Y-%m-%d %H:%M:%S")

t0 = time.mktime(time0)

assessment = " ~|~ N"

counter = 0

for i in range(i0 + 1, len(patient_lines)):

line = patient_lines[i]

tokens = line.strip().split("~|~")

if tokens[3].strip() == "Aspirin" and counter == 0:

counter += 1

time1 = time.strptime(tokens[-1].strip(), "%Y-%m-%d %H:%M:%S")

t1 = time.mktime(time1)

num = get_na_number(t1 - t0)

improvement = tokens[-4].strip()

assessment = assessment + num + improvement_signs[improvement]

patient_lines[i] = line.strip() + assessment + "\n"

elif tokens[3].strip() == "Nitroglycerin" and counter > 0:

counter += 1

num = str(counter)

improvement = tokens[-4].strip()

assessment = assessment + num + improvement_signs[improvement]

patient_lines[i] = line.strip() + assessment + "\n"

def sort_by_time(patient_lines):

swapped = True

while swapped:

swapped = False

for i in range(0, len(patient_lines) - 1):

tokens0 = patient_lines[i].strip().split("~|~")

time0 = time.strptime(tokens0[-1].strip(), "%Y-%m-%d %H:%M:%S")

t0 = time.mktime(time0)

tokens1 = patient_lines[i+1].strip().split("~|~")

time1 = time.strptime(tokens1[-1].strip(), "%Y-%m-%d %H:%M:%S")

t1 = time.mktime(time1)

if t0 > t1:

temp_line = patient_lines[i]

patient_lines[i] = patient_lines[i+1]

patient_lines[i+1] = temp_line

swapped = True

for line in sys.stdin:

tokens = line.strip().split("~|~")

if tokens[0] == "'PcrMedicationKey'":

sys.stdout.write(line)

else:

patient_id = tokens[1].strip()

if patient_id != prev_patient_id:

size = len(filtered_medicines)

if len(set(patient_medicines).intersection(filtered_medicines)) >= size:

if are_all_results_valid(patient_lines):

if are_all_times_valid(patient_lines):

sort_by_time(patient_lines)

if is_an_group(patient_lines):

add_aspirin_labels(patient_lines)

elif is_na_group(patient_lines):

add_nitro_labels(patient_lines)

for line2 in patient_lines:

sys.stdout.write(line2)

sys.stdout.write("\n")

patient_lines = []

patient_medicines = []

patient_medicines.append(tokens[3].strip())

patient_lines.append(line)

prev_patient_id = patient_id

**MANUAL FOR THE SOFTWARE**

From the NEMSIS dataset we used these databases with the python software:

- FACTPCRMEDICATION.txt, PCRVITALECGGROUP.txt, FACTPCRVITAL.txt

ECG codes (column name - eVitals_03) used in database PCRVITALECGGROUP.txt were:

9901023 Non-STEMI Anterior Ischemia

9901025 Non-STEMI Inferior Ischemia

9901027 Non-STEMI Lateral Ischemia

9901029 Non-STEMI Posterior Ischemia

9901051 STEMI Anterior Ischemia

9901053 STEMI Inferior Ischemia

9901055 STEMI Lateral Ischemia

9901057 STEMI Posterior Ischemia

First we extracted all patients with the above ECG codes located in PCRVITALECGGROUP.txt database. Then, from the resulting list we additionally extracted all patients that received Aspirin plus Nitroglycerin (located in database FACTPCRMEDICATION.txt). In order to do that we needed to combine serval databases using python software. At the end we had a list of patients with the above ECG codes who received Aspirin plus Nitroglycerin.

How to use the software:

To filter the file PCRVITALECGGROUP.txt according to needed eVitals_03 values type:

python filter1.py 9901023 9901025 9901027 9901029 9901051 9901053 9901055 9901057 <PCRVITALECGGROUP.txt >PCRVITALECGGROUP_filtered.txt

To filter FACTPCRVITAL.txt based on the VitalKey values in PCRVITALECGGROUP_filtered.txt type:

python filter2.py PCRVITALECGGROUP_filtered.txt <FACTPCRVITAL.txt >FACTPCRVITAL_filtered.txt

To filter FACTPCRMEDICATION.txt baed on the PcrKey values in FACTPCRVITAL_filtered.txt type:

python filter3.py FACTPCRVITAL_filtered.txt <FACTPCRMEDICATION.txt >FACTPCRMEDICATION_filtered.txt

To filter FACTPCRMEDICATION_filtered.txt based on the additional algorithms to generate the codes type:

python filter4.py Aspirin Nitroglycerin <FACTPCRMEDICATION_filtered.txt >FACTPCRMEDICATION_filtered2.txt
