# Supplementary material for: The timing of administering aspirin and nitroglycerin in patients with STEMI ECG changes alter patient outcome
Source: BMC Emerg Med. 2021 Nov 17;21:137. doi: 10.1186/s12873-021-00523-2 (PMC8597308; doi:10.1186/s12873-021-00523-2)

In order to exclude that this trend of reduced nitroglycerin administration in later groups is substituted by pain medications we also tested administration of pain medications. We measured only pain medications given after the first nitroglycerin in order to better correlate between them. Patients received 3 types of pain medications:

- Morphine – 497 given
- Fentanyl – 302 given
- Midazolam – 7 given.

Descriptive data of this analysis are presented in Table 4

|  | **Pts. that**  **received  opioids** | **Total drugs**  **given** | **Total No.**  **of  patients** |
| --- | --- | --- | --- |
| **A1** | 82 | 137 | 465 |
| **A2** | 70 | 109 | 308 |
| **A3** | 42 | 60 | 224 |
| **A4** | 32 | 44 | 216 |
| **A5** | 32 | 53 | 174 |
| **A6** | 35 | 56 | 210 |
| **A7** | 32 | 57 | 143 |
| **A8** | 30 | 50 | 187 |
| **A9** | 56 | 78 | 319 |
| **N1** | 41 | 62 | 234 |
| **N2** | 6 | 12 | 26 |
| **N3** | 5 | 7 | 25 |
| **N4** | 1 | 1 | 10 |
| **N5** | 16 | 23 | 53 |

**Table 4**

Graphic presentation in opioids given is presented in Figure 4. It is evident that total number of patients that received opioid has very weak downward slope of -0.18 (with R2 0.036) from A1 to A9. However, we designed a coefficient of opioids to include the number of opioids given per group. The need to give opioids is directly proportional to the number of patients that received opioids and the number of opioids that they received and is inversely proportional by the number of patients that are in the group. This can mathematically be presented as coefficient:

Coefficient of opioids = (Pts. that received opioids x Opioids given) / Total number of patients

This coefficient of opioids regards the number of patients that received opioids and the number of opioids they received. This coefficient has significant downward trend line with slope of 1.44 with relatively significant R2 of 0.34. This shows that in later groups smaller percentage of opioids “load” was needed. However these data have insignificant chi square of independence (x^2^ (16)=18.1, p=0.316). This means that giving opioids is not dependent by the previous nitroglycerin’s given nor have any dependence between the groups. In other words, giving opioids did not influence the administration of nitroglycerin in the patients.(**See supplementary 10**)

N-first group had insignificant number of opioids given and is not presented.

**
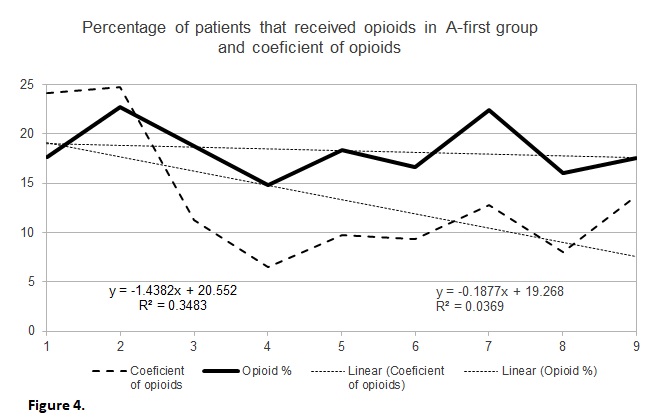
**

In order to find some reason why some patient’s received nitroglycerin sooner after aspirin and some later (A1 through A9) or why some patients received nitroglycerin first we analyzed a number of vital signs and demographic data recorded in the NEMSIS dataset **(See supplementary 4,5)**. We tested the averages of:

- Systolic blood pressure (SBP),
- Heart rate,
- Pulse oximetry findings
- Pain severity on a scale from 1 to 10.
- Type of ischemia (STEMI or NON-STEMI)
- Age
- Gender differences

Descriptive data for these analyses are presented in Table 5.

| **A-FIRST GROUP** | A1 | A2 | A3 | A4 | A5 | A6 | A7 | A8 | A9 | | |
| --- | --- | --- | --- | --- | --- | --- | --- | --- | --- | --- | --- |
| SBB (mmHg) | 147.01 | 144.57 | 141.65 | 148.57 | 143.87 | 141.13 | 140.02 | 136.69 | 140.75 |  |  |
| Heart rate | 86.16 | 84.55 | 84.39 | 83.25 | 84.65 | 83.90 | 87.17 | 83.40 | 82.08 |  |  |
| SpO2 | 96.02 | 96.79 | 96.14 | 96.86 | 96.15 | 96.25 | 95.96 | 95.78 | 96.66 |  |  |
| Respirations | 18.09 | 18.78 | 19.13 | 18.84 | 20.44 | 19.13 | 19.27 | 18.90 | 18.58 |  |  |
| Pain scale | 6.08 | 6.56 | 6.55 | 6.01 | 6.08 | 6.08 | 6.12 | 6.47 | 5.94 |  |  |
| STEMI | 431.00 | 286.00 | 211.00 | 198.00 | 161.00 | 196.00 | 134.00 | 173.00 | 298.00 |  |  |
| NON-STEMI | 33.00 | 22.00 | 13.00 | 18.00 | 13.00 | 14.00 | 9.00 | 14.00 | 28.00 |  |  |
| % STEMI | 92.89 | 92.86 | 94.20 | 91.67 | 92.53 | 93.33 | 93.71 | 92.51 | 91.41 |  |  |
| % NON-STEMI | 7.11 | 7.14 | 5.80 | 8.33 | 7.47 | 6.67 | 6.29 | 7.49 | 8.59 |  |  |
| MALES | 329.00 | 213.00 | 157.00 | 144.00 | 121.00 | 152.00 | 91.00 | 135.00 | 209.00 |  |  |
| FEMALES | 135.00 | 94.00 | 66.00 | 72.00 | 53.00 | 58.00 | 52.00 | 50.00 | 110.00 |  |  |
| AGE | 60.88 | 61.52 | 63.68 | 63.23 | 62.76 | 62.78 | 62.90 | 64.77 | 62.87 |  |  |
| %MALE | 70.91 | 69.38 | 70.40 | 66.67 | 69.54 | 72.38 | 63.64 | 72.97 | 65.52 |  |  |
| %FEMALE | 29.09 | 30.62 | 29.60 | 33.33 | 30.46 | 27.62 | 36.36 | 27.03 | 34.48 |  |  |
| RATIO M/F | 2.44 | 2.27 | 2.38 | 2.00 | 2.28 | 2.62 | 1.75 | 2.70 | 1.90 |  |  |
|  |  |  |  |  |  |  |  |  |  |  |  |
| **N-FIRST GROUP** | N1 | N2 | N3 | N4 | N5 |  |  |  |  |  |  |
| SBB (mmHg) | 144.03 | 142.40 | 130.95 | 132.60 | 149.19 |  |  |  |  |  |  |
| Heart rate | 85.37 | 85.90 | 93.00 | 79.00 | 96.00 |  |  |  |  |  |  |
| SpO2 | 96.56 | 93.25 | 94.79 | 87.00 | 94.94 |  |  |  |  |  |  |
| Respirations | 19.45 | 25.03 | 18.64 | 17.80 | 21.14 |  |  |  |  |  |  |
| Pain scale | 6.19 | 4.69 | 5.16 | 4.44 | 6.25 |  |  |  |  |  |  |
| STEMI | 214.00 | 26.00 | 24.00 | 9.00 | 50.00 |  |  |  |  |  |  |
| NON-STEMI | 20.00 | 0.00 | 1.00 | 1.00 | 3.00 |  |  |  |  |  |  |
| % STEMI | 91.45 | 100.00 | 96.00 | 90.00 | 94.34 |  |  |  |  |  |  |
| % NON-STEMI | 8.55 | 0.00 | 4.00 | 10.00 | 5.66 |  |  |  |  |  |  |
| MALES | 163.00 | 18.00 | 18.00 | 8.00 | 31.00 |  |  |  |  |  |  |
| FEMALES | 70.00 | 8.00 | 7.00 | 2.00 | 22.00 |  |  |  |  |  |  |
| AGE | 65.06 | 66.76 | 68.08 | 59.80 | 67.05 |  |  |  |  |  |  |
| %MALE | 69.96 | 69.23 | 72.00 | 80.00 | 58.49 |  |  |  |  |  |  |
| %FEMALE | 30.04 | 30.77 | 28.00 | 20.00 | 41.51 |  |  |  |  |  |  |
| RATIO M/F | 2.33 | 2.25 | 2.57 | 4.00 | 1.41 |  |  |  |  |  |  |

**Index for table 5:**

SBB (mmHg) – average systolic blood pressure in mmHg.

Heart rate – average heart rate

SpO2 – average SpO2

Respirations – average respirations per minute

Pain scale – average pain on a scale from 1 to 10

STEMI – total number of patients with STEMI

NON-STEMI – total number of patients with NON-STEMI

% STEMI – percentage of STEMI patients

% NON-STEMI – percentage of NON-STEMI pacientis

MALES – total number of males

FEMALES – total number of females

AGE – average age of patents

%MALE – percentage of males

%FEMALE – percentage of females

RATIO M/F – ratio between males and females

**Table 5**

No significant correlation was found for any of the vital signs except for systolic blood pressure and age of the patients in A-group as presented on Figure 4 and Figure 5.

**
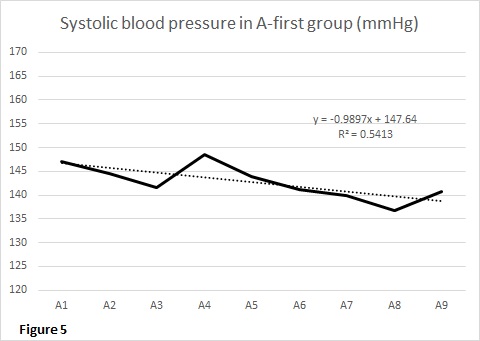
**

Figure 5 shows down sloping trend line with slope of 0.99 (r2 of 0.54) which show that on average A9 group had 8.9 mmHg lower systolic blood pressure compared to A1 group and this is consistent trend from A1 to A9 group. In some patient’s systolic blood pressure was measured several times and we only compared the average for the whole group. Rationale behind this decision was to find a pattern that can explain the postponing of nitroglycerin administration. Therefore, systolic blood pressure may explain why nitroglycerin was given later in these groups since low systolic blood pressure is most important contraindication for administering nitroglycerin in acute coronary syndrome.

We found another significand trend in A-first group for the average age. There is up-sloping trend (slope 0.26 with R2 of 0.4) for the age of the patients from A1 to A9 group as presented in Figure 6. This means older patients received nitroglycerin later in A-group. We cannot identity any rationale for this trend, but age may have had some effect on the decision to postpone the administration of the first dose of nitroglycerin.


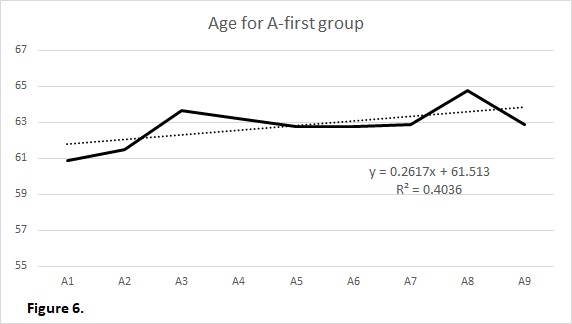


No similar correlations and significant trends were found in N-group.

We analyzed what type of ACS was most prevalent within different groups in the study and the result showed that more than 90% in both groups were STEMI myocardial ischemia, and less than 10% were NON_STEMI as shown in figure 7. There was no significant difference between different groups and the spread of this tendency is consistent in all groups. This means most of the patients had more severe type of acute coronary syndrome, and medical personnel was hesitant in administering nitroglycerin in some patients. Meaning medical personal is administering nitroglycerin only in most severe patients with STEMI ischemia and relatively higher systolic blood pressure.


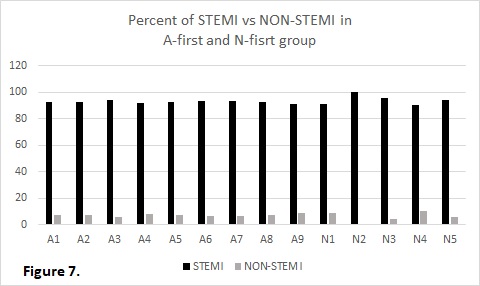

Supplement: Supplementary file 11 — Additional file 11. [file 12873_2021_523_MOESM11_ESM.docx]
